# Biology of Heme in Mammalian Erythroid Cells and Related Disorders

**DOI:** 10.1155/2015/278536

**Published:** 2015-10-18

**Authors:** Tohru Fujiwara, Hideo Harigae

**Affiliations:** ^1^Department of Hematology and Rheumatology, Tohoku University Graduate School of Medicine, Sendai 980-8575, Japan; ^2^Molecular Hematology/Oncology, Tohoku University Graduate School of Medicine, Sendai 980-8575, Japan

## Abstract

Heme is a prosthetic group comprising ferrous iron (Fe^2+^) and protoporphyrin IX and is an essential cofactor in various biological processes such as oxygen transport (hemoglobin) and storage (myoglobin) and electron transfer (respiratory cytochromes) in addition to its role as a structural component of hemoproteins. Heme biosynthesis is induced during erythroid differentiation and is coordinated with the expression of genes involved in globin formation and iron acquisition/transport. However, erythroid and nonerythroid cells exhibit distinct differences in the heme biosynthetic pathway regulation. Defects of heme biosynthesis in developing erythroblasts can have profound medical implications, as represented by sideroblastic anemia. This review will focus on the biology of heme in mammalian erythroid cells, including the heme biosynthetic pathway as well as the regulatory role of heme and human disorders that arise from defective heme synthesis.

## 1. Heme Biosynthesis

**Figure 1:**
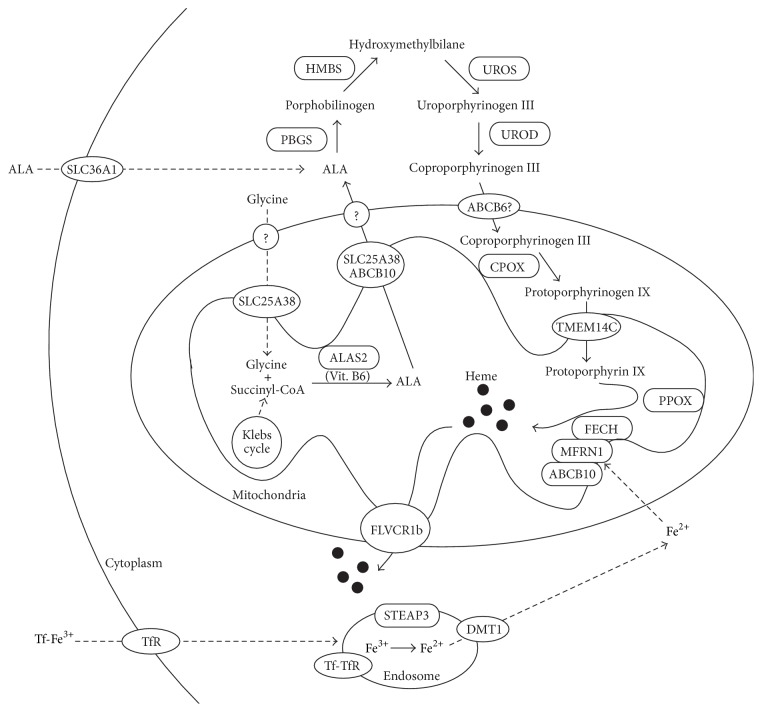
Heme biosynthetic pathway in erythroid cells. Schematic representation of the heme biosynthetic pathway in erythroid cells. Heme synthesis begins with the condensation of glycine and succinyl-CoA to form ALA. Next, ALA is transported outside of the mitochondria and catalyzed to form coproporphyrinogen III. CPOX converts coproporphyrinogen III to protoporphyrinogen IX, which is subsequently oxidized into protoporphyrin IX by PPOX. Finally, ferrous iron is incorporated into protoporphyrinogen IX to form heme in a reaction catalyzed by FECH. FECH is localized in the inner mitochondrial membrane and associates with MFRN1 and ABCB10. SLC25A38 and ABCB10 have been proposed as mitochondrial ALA exporters located on the inner mitochondrial membrane. ABCB6 and TMEM14C have been proposed as putative coproporphyrinogen III and protoporphyrinogen IX importers, respectively. FLVCR1b is a mitochondrial heme exporter. Tf-bound Fe^3+^ is bound to TfR, released into endosome, and reduced to Fe^2+^ by STEAP3. Subsequently, Fe^2+^ exits the endosome via DMT1 and enters the mitochondria via MFRN1. ALAS2: erythroid-specific *δ*-aminolevulinate synthase, ALA: *δ*-aminolevulinic acid, PBGS: porphobilinogen synthase, HMBS: hydroxymethylbilane synthase, UROS: uroporphyrinogen synthase, UROD: uroporphyrinogen decarboxylase, CPOX: coproporphyrinogen oxidase, PPOX: protoporphyrinogen IX oxidase, FECH: ferrochelatase, MFRN1: mitoferrin 1, Vit. B6: vitamin B6, SLC25A38: solute carrier family 25 member 38, ABCB10: ATP-binding cassette subfamily B member 10, TMEM14C: transmembrane protein 14C, FLVCR1b: feline leukemia virus subgroup C receptor, Tf: transferrin, TfR: transferrin receptor, STEAP3: six-transmembrane epithelial antigen of prostate 3, and DMT1: divalent metal transporter 1. Adapted and modified from [12–14].

The mammalian heme biosynthetic pathway includes eight enzymes (reviewed in [1–5]) (Figure 1). The first and rate-limiting step of this pathway is the condensation of glycine and succinyl-CoA to form 5-aminolevulinic acid (ALA), a 5-carbon aminoketone, in the mitochondrial matrix. This reaction is catalyzed by ALA synthase (ALAS). ALAS has two isozymes encoded by the housekeeping and erythroid-specific ALAS2 genes, which are termed ALAS1 (or ALAS-N) and ALAS2 (or ALAS-E), respectively. The human genes encoding ALAS1 and ALAS2 have been mapped on 3p21.1 [6] and Xp11.21 [7], respectively. Whereas heme negatively regulates ALAS1 expression by directly binding to a CP motif (described later) [1], ALAS2 expression is strongly induced during the later stage of erythroid differentiation. The regulatory region of the* ALAS2* gene contains transcription factor-binding elements such as CCAAT, TATA, GATA, CACCC, Sp1, and GATA-1 that are thought to induce erythroid-specific expression of the* ALAS2* gene [8, 9]. Furthermore, the 5′-untranslated region (UTR) of* ALAS2* contains an iron-responsive element (IRE) that interacts with iron-responsive proteins (IRPs), thereby regulating ALAS2 expression at the posttranscriptional level. Under conditions of iron deficiency, ALAS2 translation is inhibited by the binding of IRPs to the IRE; in contrast, IRPs detach from the IRE under conditions of iron sufficiency, resulting in increased ALAS2 translation (for the IRP-IRE system, described later).

ALA is exported to the cytosol, where two molecules of ALA are condensed into the monopyrrole porphobilinogen (PBG), which is generated by PBG synthase (PBGS). The PBGS crystal structure is homo-octomeric, and each monomer binds one zinc atom to exert its enzymatic activity [10]. In addition, both housekeeping and erythroid-specific* PBGS* mRNAs have been detected. However, these mRNAs differ only in the 5′-UTR, and thus the housekeeping and erythroid-specific forms of the PBGS enzyme are identical [11].

Four PBG molecules are joined by hydroxymethylbilane synthase (HMBS) to form the first cyclic tetrapyrrole HMB, which is then converted to uroporphyrinogen III by uroporphyrinogen synthase (UROS). Uroporphyrinogen III is subsequently decarboxylated by uroporphyrinogen decarboxylase (UROD) to form coproporphyrinogen III. Similar to* PBGS*, both housekeeping and erythroid-specific mRNAs exist for* HMBS* and* UROS*. The housekeeping HMBS contains an additional 17 amino acid residues at the amino terminus as compared to the erythroid form of the protein; in contrast, the housekeeping and erythroid UROS proteins are identical.

Coproporphyrin III enters the mitochondria, where it is oxidatively decarboxylated by coproporphyrinogen oxidase (CPOX) to form protoporphyrinogen IX. Protoporphyrinogen IX is then oxidized to protoporphyrin IX by protoporphyrinogen oxidase (PPOX). Finally, ferrous iron is inserted into protoporphyrin IX by ferrochelatase (FECH) to form heme. FECH is another rate-limiting enzyme of the heme biosynthetic pathway. This enzyme exists as a homodimer in which each subunit contains a [2Fe-2S] cluster that is necessary for the enzymatic activity [15]. In addition,* FECH* expression is induced during erythroid differentiation, wherein it is controlled by the Sp1, NF-E2, and GATA elements [16].

Heme biosynthesis, therefore, also relies on the intracellular availability of iron. In erythroid cells, iron is acquired via transferrin receptor-mediated endocytosis of circulating transferrin-iron (III) (Fe^3+^) complexes. Once internalized, transferrin-bound Fe^3+^ is released, reduced to Fe^2+^ by six-transmembrane epithelial antigen of the prostate 3 (STEAP3) [17], exits the endosome via divalent metal transporter 1 (DMT1), and subsequently enters the mitochondria. The inner mitochondrial membrane protein mitoferrin 1 (MFRN1) plays an important role in supplying iron for heme biosynthesis as well as iron-sulfur clusters in the mitochondria [18]. MFRN1 is responsible for iron transport into the mitochondria, although the presence of another inner membrane protein, ABCB10, is required to stabilize MFRN1 [19]. Although the precise role of ABCB10 has yet to be elucidated, MFRN1 forms a complex with FECH and ABCB10 that might allow the direct transfer of ferrous iron for heme and/or iron-sulfur cluster synthesis [20].

## 2. Transport of Heme and Porphyrin Intermediates

As described above, heme biosynthetic enzymes are fairly well understood. However, relatively less is known about the transport of heme and porphyrin intermediates. The following describes the recent understanding regarding the transport of heme and porphyrin intermediates (Figure 1).

Glycine is required for the first step of porphyrin synthesis and must be transported from the cytosol into the mitochondria. Solute carrier family 25 member 38 (SLC25A38) was recently identified through the positional cloning of a gene implicated in nonsyndromic congenital sideroblastic anemia [21]. Yeast lacking the* SLC25A38* ortholog YDL119c exhibits a defect in ALA biogenesis [21], suggesting that SLC25A38 is required for glycine import. The ability of SLC25A38 and ABCB10 to export ALA from mitochondria has also been proposed (Figure 1) [3]. Because both SLC25A38 and ABCB10 are located in the inner mitochondrial membrane, it remains to be elucidated how ALA could be exported through the outer membrane. However, extracellular ALA can be supplied across the plasma membrane for heme biosynthesis. Among the ALA transporters, SLC36A1 is abundantly expressed in erythroid cells (Figure 1) [22]. However, the significance of extracellular ALA with respect to erythropoiesis and normal erythroid differentiation remains to be elucidated.

In the cytosol, ALA is converted to coproporphyrinogen III, which is subsequently transported to the mitochondrial intermembrane space. Several studies have suggested that adenosine triphosphate- (ATP-) binding cassette, subfamily B member 6 (ABCB6), which is located in the mitochondrial outer membrane, is responsible for coproporphyrinogen III transport [23, 24]. However, these findings remain controversial because ABCB6 has also been detected in the plasma membrane, Golgi, and lysosomes [25–27] and is even used to define the blood group Langereis [28]. In addition, a defect in ABCB6 can cause inherited developmental defects of the eye (ocular coloboma) [29] and skin (dyschromatosis universalis hereditaria) [30]. Nevertheless, ABCB6 expression is obviously induced during erythroid differentiation [3, 4, 31], suggesting that it may play an important role in erythropoiesis and indicating a need for further analyses.

Recently, a large-scale screening was conducted to identify mitochondrial proteins that are coexpressed with the core machinery of heme biosynthesis [31]. One of these genes, transmembrane protein 14C (TMEM14C), encodes the mitochondrial inner membrane protein, which is essential for erythropoiesis in zebrafish and mice [12, 31]. Functional analyses have suggested the involvement of TMEM14C in the import of protoporphyrinogen IX into the mitochondrial matrix for heme synthesis and subsequent hemoglobin production [12]. Despite its essential role during erythropoiesis, human disorders resulting from defects in TMEM14C have not yet been identified. Another mitochondrial gene, solute carrier family 25 member 39 (SLC25A39), is required for heme synthesis in yeast, zebrafish, and mammalian erythroid cells [31]. However, the exact role of SLC25A39 in mammalian erythropoiesis requires further investigation.

In mammals, the membrane protein feline leukemia virus, subgroup C receptor 1, SLC49A1 (FLVCR1) was recently identified as a heme exporter. There are two different isoforms of FLVCR1, FLVCR1a and FLVCR1b, which are expressed on the plasma membrane and the mitochondria, respectively [32–34]. Recent studies have revealed that the mitochondrial heme exporter FLVCR1b, which contains shortened N-terminus resulting from an alternative transcriptional start site (in contrast to originally identified FLVCR1a), is essential for erythroid differentiation [34]. On the other hand, targeted disruption of* Flvcr1a* resulted in skeletal defects and vascular abnormalities but not anemia, implying that FLVCR1a is dispensable for erythropoiesis [3]. A closely related homologue, FLVCR2 (SLC49A2), was reported to be ubiquitously expressed membrane-attached heme importer [35]. However, the physiological roles of FLVCR2 in erythropoiesis remain to be elucidated [5].

## 3. Heme Is Involved in Several Biological Processes during Erythroid Differentiation

In addition to acting as a prosthetic group in hemoproteins such as hemoglobin, heme itself might affect several biological processes during erythroid differentiation, including transcription, translation, protein degradation, and microRNA (miRNA) processing [1, 36, 37]. Heme modifies the function or localization of its target proteins through direct binding to CP motifs, which consist of cysteine and proline residues [1, 36, 37]. CP motif-containing proteins, namely, heme-binding proteins, reportedly include heme activator protein 1 (Hap1), BTB and CNC homology 1, basic leucine zipper transcription factor 1 (BACH1) and BACH2, ALAS1, ALAS2, heme oxygenase 2 (HO-2), iron-responsive element-binding protein 2 (IRP2), heme-oxidized IRP2 ubiquitin ligase-1 (HOIL-1), and heme regulated inhibitor (HRI) [37, 38].

BACH1 is a basic leucine zipper transcriptional repressor that can bind members of the small Maf family through its Maf recognition element (MARE) to form heterodimers [37, 39]. Binding of heme to CP motifs within BACH1 inhibits the DNA-binding ability of BACH1, induces its dissociation from small Maf proteins, triggers its export from the nucleus, and induces its ubiquitination and degradation [39–42]. Therefore, whereas the BACH1-small Maf heterodimer formed via MARE site binding suppresses target gene transcription, heme displaces BACH1, allowing small Maf to heterodimerize with nuclear factor, erythroid 2 (p45 NF-E2), or nuclear factor, erythroid 2-like 2 (NRF2) via the MARE site, resulting in transcriptional activation [37]. In erythroid cells, BACH1 directly regulates the expression of globins [43] and HO-1 [44], suggesting an important role for BACH1 in erythroid differentiation as well as heme metabolism. Beyond erythroid cells, BACH1 also regulates the expression of ferroportin [45], ferritins [46], and SPI-C, which promotes monocyte differentiation to iron-recycling macrophages [47], and thus suggests a role for BACH1 in systemic iron homeostasis. Other heme regulated transcriptional regulators include* Drosophila melanogaster* E75 and its mammalian homologue Rev-Erb*α*/*β*, both of which belong to the nuclear hormone receptor superfamily and can bind heme via their respective ligand-binding domains [37, 48, 49].

Heme also controls target gene translation by binding to the two heme-binding domains of HRI, a known protein kinase [50]. HRI is autophosphorylated at multiple sites under conditions of heme deficiency, an essential process for kinase activity [51, 52]. Under conditions of heme deficiency, HRI inhibits mRNA translation by phosphorylating the *α*-subunit of the eukaryotic initiation factor (eIF2*α*) [50], whereas an increased heme concentration during erythroid differentiation inhibits HRI activity, thereby promoting the translation of *α*- and *β*-globins [53, 54]. Developing erythroid cells synthesize enormous amounts of hemoglobin but must synthesize the correct amounts of globin proteins and heme because of the intrinsic toxicities of these molecules; for example, excess globins cause proteotoxicity [54, 55], whereas free heme is a potent oxidative molecule that can produce reactive oxygen species (ROS) via the Fenton reaction [56]. Therefore, it appears reasonable to consider that heme contributes to the coordination of globin and heme syntheses.

As previously described, heme participates in transcriptional regulation through binding to BACH1 and inducing its ubiquitination and subsequent degradation [37, 42], which can be considered another regulatory action of heme. In addition, the IRP-IRE system has been shown to posttranscriptionally regulate the expression of several mRNAs, including those encoding ALAS2, transferrin receptor, ferroportin, DMT1, and ferritin, thus playing an important role in iron homeostasis (reviewed in [1, 57]). IRPs comprise two independent proteins, IRP1 and IRP2, the latter of which predominantly regulates iron homeostasis* in vivo* [58]. Interestingly, heme binds to IRP2 through the CP motif within the iron dependent degradation (IDD) domain to induce its ubiquitination/degradation [59, 60]. Therefore, the regulatory processes of heme biosynthesis and iron metabolism in erythroid cells appear to be closely related.

Finally, recent reports have revealed that heme also regulates the processing of a class of noncoding RNAs (ncRNAs) that includes housekeeping RNAs (rRNAs, tRNAs, and snoRNAs), small and long ncRNAs (<200 and >200 bases, resp.), small interfering RNAs (siRNAs), PIWI-interacting RNAs (piRNAs), and miRNAs [61]. miRNAs are now universally recognized as an extensive and ubiquitous class of regulatory molecules [62]. Heme binds to the cysteine residue at amino acid position of 352 of the RNA-binding protein DiGeorge critical region 8 (DGCR8) and induces the dimerization of DGCR8, thereby promoting the processing of pri-miRNA into mature miRNA [63, 64]. Whereas the role of miRNAs during erythroid differentiation is well understood [65], the significance of heme-mediated miRNA processing during erythroid differentiation remains to be elucidated and thus requires further investigation.

## 4. Disorders Resulting from Defective Heme Biosynthesis in Erythroid Cells

Heme is mainly produced by erythroblasts and hepatocytes. Whereas heme negatively regulates the expression of ALAS1 via a feedback mechanism in nonerythroid cells (hepatocytes) [1], it does not negatively regulate ALAS2 in erythroblasts; in fact, heme can promote its own synthesis by regulating ALAS2 expression through the IRP-IRE system. Therefore, the regulation of heme biosynthesis should be considered separately in erythroid and nonerythroid cells. In addition, a defect in any heme biosynthetic enzyme does not result in uniform systematic consequences [2]. For example, defects in PBGS or HMBS impair heme biosynthesis in the liver, leading to the onset of ALA dehydratase deficiency porphyria (ADP) or acute intermittent porphyria (AIP), respectively, whereas no abnormality is observed in erythroblasts. On the other hand, defects in FECH or UROS do not severely impair heme biosynthesis in the liver but instead result in the onset of erythropoietic protoporphyria (EPP) or congenital erythropoietic porphyria (CEP). Accordingly, each disorder is referred to as either hepatic or erythropoietic porphyria.

This review mainly focuses on hematologically relevant human disorders resulting from defects in heme biosynthesis, such as sideroblastic anemia and erythropoietic porphyria.

### 4.1. Sideroblastic Anemia

Sideroblastic anemias are a heterogenous group of syndromic and nonsyndromic disorders with the common features of mitochondrial iron accumulation in bone marrow erythroid precursors (ring sideroblasts), ineffective erythropoiesis, increased tissue iron levels, and varying proportions of hypochromic erythrocytes in the peripheral blood [13, 66, 67]. Whereas these syndromes are commonly acquired and predominantly associated with myelodysplastic syndrome, of which a significant portion of cases might result from a mutation in the RNA splicing machinery componentsplicing factor 3b, subunit 1 (*SF3B1*) [68], congenital forms of sideroblastic anemia (CSA) have been reported; it is a rare and heterogeneous disease caused by mutations of genes involved in heme biosynthesis (*ALAS2*,* SLC25A38*), iron-sulfur [Fe-S] cluster biosynthesis (*ABCB7*,* GLRX5*), iron reduction (*STEAP3*), and mitochondrial protein synthesis (mitochondrial DNA,* PUS1*,* YARS2*,* TRNT1*, and* SLC19A2*) (described in [20, 66, 67, 69–72]) (Table 1).

#### 4.1.1. X-Linked Sideroblastic Anemia

The most common form of CSA is XLSA (X-linked sideroblastic anemia), which is attributed to mutations in the X-linked gene* ALAS2* [1, 73]. Defects in ALAS2 result in decreased protoporphyrin synthesis and subsequent reductions in iron incorporation and heme synthesis, leading to microcytic anemia and the appearance of ring sideroblasts in the bone marrow. Typically, XLSA patients are male and present before the age of 40 years; however, this disorder occurs across a broad range of ages and can affect elderly patients [74]. On the other hand, anemia may also present in heterozygous female carriers, presumably because of skewed X inactivation or the excessive age-related skewing that occurs in hematopoietic tissues [75, 76].

Most of the XLSA-associated mutations in* ALAS2* are missense substitutions resulting in loss of functionality [20, 67, 69, 77, 78], whereas mutations in the* ALAS2* regulatory region, such as the promoter [79] and intron 1 [8, 9, 80], lead to decreased* ALAS2* expression. ALAS2 missense mutations commonly decrease the binding of pyridoxal 5′-phosphate (PLP, vitamin B6), which is a cofactor for ALAS2 enzymatic activity, thus accounting for the PLP responsiveness in XLSA patients carrying such mutations [7, 81]. However, nearly half of XLSA cases are unresponsive to PLP [13, 82]. In such cases, ALA supplementation might mitigate these impairments [22]. In our recent preclinical* in vitro* analysis, ALA restores defects in ALAS2 deficiency based on human induced pluripotent stem (iPS) cell-derived erythroblasts [22]. Because ALA is an endogenous amino acid that has been shown to be safe in clinical settings [83], it might be considered to administer ALA in patients with XLSA who are refractory to PLP supplementation.

### 4.2. Erythropoietic Porphyria

Erythropoietic porphyria includes both erythropoietic protoporphyria and congenital erythropoietic porphyria. The former comprises two variants, erythropoietic protoporphyria (EPP) and X-linked protoporphyria (XLPP), and is classified among the cutaneous porphyrias characterized by the accumulation of protoporphyrin IX, which results in photosensitivity. EPP and XLPP are clinically indistinguishable but result from the mutations of different genes:* FECH* and* ALAS2*, respectively.

#### 4.2.1. Erythropoietic Protoporphyria (EPP)

EPP is an inherited disorder caused by a partial deficiency in FECH activity [2, 84]. In this disorder, protoporphyrin accumulates in the erythroblasts and is subsequently taken up by the liver and skin. Cutaneous photosensitivity characteristically begins in childhood in the absence of neurological involvement [2, 84]. Liver damage is observed in patients with severe symptoms [84]. In addition, hypochromic microcytic anemia occurs in 20–60% of cases [85], and ring sideroblasts may be observed [86]. Elevated levels of erythrocyte protoporphyrin are observed. However, in contrast to iron deficiency anemia and lead poisoning, in which the increased erythrocyte protoporphyrin is chelated with zinc, erythrocytic protoporphyrin in EPP remains in a free state [87].

The inheritance of EPP is considered autosomal recessive but is a bit unusual. In most patients, EPP results from the coinheritance of a specific FECH mutation* in trans* with a hypomorphic low-expression FECH polymorphism (IVS3-48C) [88]. The IVS3-48C allele of this polymorphism increases the use of an aberrant splicing site that may contribute to disease onset [89]. Ethnic differences have been reported in the frequency of the IVS3-48C allele; specifically, the frequency of this allele differs widely among Japanese (43%), southeast Asian (31%), white French (11%), North African (2.7%), and black West African (<1%) populations [89].

Besides avoiding exposure to sunlight, the treatment for EPP includes the oral administration of *β*-carotene and afamelanotide, transfusion of cholestyramine red cells, and, in severe cases, liver transplantation [2, 90]. Low-dose intravenous iron therapy may also be effective [91].

#### 4.2.2. X-Linked Protoporphyria (XLPP)

XLPP is a clinically indistinguishable X-linked form of EPP caused by gain-of-function mutations in* ALAS2* [92]. This gain-of-function of ALAS2 increases protoporphyrin IX production in the context of normal FECH activity, the latter of which becomes rate-limiting and presumably leads to the onset of the EPP-like disease phenotype. In contrast to EPP, in XLPP the erythrocyte levels of both zinc protoporphyrin and free protoporphyrin are increased. This disease is similar but not identical to that described in* Irp2* ^−/−^ mice, which are characterized by the overexpression of ALAS2 consequent to the loss of IRP2-dependent translational repression [93].

Mutations of* ALAS2* include c.1706-1709 delAGTG (p.E569GfsX24) and c1699-1700 delAT (p.M567EfsX2), which result in frameshifts that cause replacement or deletion of 19-20 carboxy-terminal residues from ALAS2 [93]. Recently, the novel* ALAS2* mutations c.1734 delG and c.1642 C >T (p.Q548X) have been identified [94]. The treatment of XLPP is similar to that of the biochemically and phenotypically similar EPP.

#### 4.2.3. Congenital Erythropoietic Porphyria (CEP)

CEP is an autosomal recessive erythropoietic porphyria characterized by severe photosensitivity and hemolytic anemia [2]. This disease results from a mutation in the* UROS* gene, and recent work has suggested that an accompanying gain-of-function mutation in ALAS2 could modify the disease severity [95]. In addition, an X-linked form of CEP has been reported in which a trans-acting GATA-1-R216W mutation contributes to the onset of CEP [96].

## 5. Conclusion

Elucidation of heme biology in the mammalian erythroid cell would provide an important insight to understand and to get more efficient targets for the human disorders that arise from defective heme synthesis.

## Figures and Tables

**Table 1:** Genetic features of congenital sideroblastic anemias.

	Inheritance	Chromosome	Gene	Treatment
XLSA	X-linked	Xp11.21	ALAS2	Vitamin B6
XLSA/A	X-linked	Xp13.3	ABCB7	—
SA/GLRX5	Recessive	14q32.13	GLRX5	?
SA/SLC25A38	Recessive	3p22.1	SLC25A38	?
SA/STEAP3	?	2q14.2	STEAP3	—
PMPS	Maternal^*∗*^	Mitochondria	Mitochondrial	—
TRMA	Recessive	1q24.2	SLC19A2	Thiamine
MLASA/PUS1	Recessive	12q24.33	PUS1	—
MLASA/YARS2	Recessive	12p11.21	YARS2	—
SIFD/TRNT1	Recessive	3p26.2	TRNT1	—

XLSA: X-linked sideroblastic anemia; XLSA/A: X-linked sideroblastic anemia with ataxia; PMPS: Pearson Marrow Pancreas Syndrome; TRMA: thiamine-responsive megaloblastic anemia; MLASA: mitochondrial myopathy and sideroblastic anemia. SIFD: syndromic form of congenital sideroblastic anemia associated with B-cell immunodeficiency, periodic fevers, and developmental delay. Adopted and modified from [13, 14]. ^*∗*^Sporadic cases are also observed.
